# Enhancement of electroluminescence from embedded Si quantum dots/SiO_2_multilayers film by localized-surface-plasmon and surface roughening

**DOI:** 10.1038/srep11881

**Published:** 2015-07-03

**Authors:** Wei Li, Shaolei Wang, Mingyue Hu, Sufeng He, Pengpeng Ge, Jing Wang, Yan Yan Guo, Liu Zhaowei

**Affiliations:** 1 College of Electronic Science and Engineering, Nanjing University of Posts and Telecommunications, Nanjing, 210003 China; 2 Key Laboratory of Radio Frequency and Micro-Nano Electronics of Jiangsu Province, Nanjing 210023, Jiangsu, China; 3 Department of Electrical and Computer Engineering, University of California, San Diego, 9500 Gilman Drive, La Jolla, California 92093-0407, USA

## Abstract

In this paper, we prepared a novel structure to enhance the electroluminescence intensity from Si quantum dots/SiO_2_multilayers. An amorphous Si/SiO_2_ multilayer film was fabricated by plasma-enhanced chemical vapor deposition on a Pt nanoparticle (NP)-coated Si nanopillar array substrate. By thermal annealing, an embedded Si quantum dot (QDs)/SiO_2_ multilayer film was obtained. The result shows that electroluminescence intensity was significantly enhanced. And, the turn-on voltage of the luminescent device was reduced to 3 V. The enhancement of the light emission is due to the resonance coupling between the localized-surface-plasmon (LSP) of Pt NPs and the band-gap emission of Si QDs/SiO_2_ multilayers. The other factors were the improved absorption of excitation light and the increase of light extraction ratio by surface roughening structures. These excellent characteristics are promising for silicon-based light-emitting applications.

It is well known silicon is an indirect bandgap material. It is not a good choice for light emitting applications. But, it is highly desired to develop Si-based LED materials because of their ease in integrating with the already existing Si integrated circuit technology. And another reason is obviously cost considerations. The other materials, such as GaN, carbon nanotubes and grapheme, are expensive for LED. Actually, the researches on silicon nanostructure luminescence began in the1990 s after the discovery of strong visible photoluminescence (PL) from porous silicon at room temperature^1^,^2^,^3^,^4^. In addation, it has been reported silicon nanosphere as one kinds of all-dielectric materials with low-loss nature and strong magnetic response can induces near-zero reflection and near-perfect transmission^5^,^6^. It will be applied in nanophotonics materials. So, it is very important to reseach the silicon nanomaterials.

Among the many kinds of Si quantum structures, embedded Si quantum dot (QDs)/SiO_2_ or embedded Si quantum dot/SiN_X_ multilayer films have attracted a lot more attention due to its potential application in metal-semiconductor-oxide light emitting diodes^7^,^8^,^9^,^10^,^11^,^12^. In contrast to porous silicon, these films can be obtained on various substrates. Their thickness can range from several nanometers to several microns, which is important in research on the electroluminescence.

Despite the huge progress in the luminescence of Si QDs-based system,it is still under investigation for use in optical interconnections in very large scale integration (VLSI). The mechanism of light emission from Si-QDs has been solved due to the observation of the quantum confinement effect (QCE)^13^,^14^. It was reported that the light emission of Si-QDs is based on the radiative recombination occurring at surface defect states surrounding the Si-QD core. Another possible principle has attributed the luminescence from the band-to-band transition with Si-QDs to QCE^15^. Because of the large difference in refractive index between Si and SiO_2_ (or SiN_X_), the emission light will be internally reflected on the interface. As a result, the fraction of external light in the Si QDs-based multilayer structures is very small. Many methods, such as introducing photonic crystal structures^16^, localized surface plasmon^17^ and surface roughening^18^ have been applied to address this problem. Localized surface plasmon is one way to enhance light emission due to coupling between the LSP of metallic NPs and the band-gap emission of materials. Surface roughening is another effective way to improve the light extraction ratio by forming the anti-reflection surface and reducing the surface reflection. In this paper, these two methods were both used to enhance the light emission. We prepared a Si QDs /SiO_2_ multilayer film on a Pt NPs-coated Si nanopillar array substrate. The Pt NPs were used for their localized surface plasmons and the nanopillar array was used for surface roughening. The result shows that electroluminescence intensity was significantly enhanced. And, the turn-on voltage of the luminescent device was reduced to 3 V.

## Experiments

### (1) Fabrication process of Si nanopillar arrays

**Figure 1:**
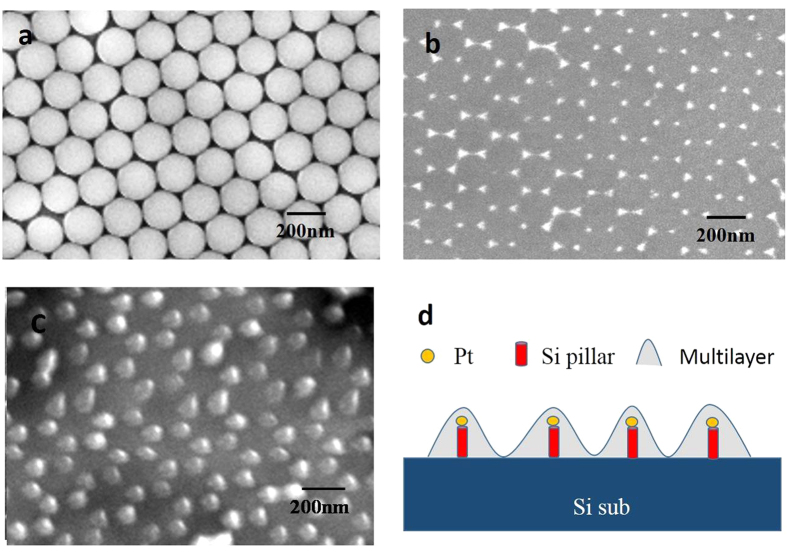
SEM image of single layer of PS spheres (**a**), Pt nanoparticles (**b**) Pt nanoparticles coated Si nanopillar (**c**) and the schematic diagram of electroluminescent structures (**d**).

The ordered Si nanopillar array substrate was fabricated by nanosphere lithography. It was reported in our previous paper^19^,^20^,^21^. In the first step, a single layer of polystyrene (PS) spheres with diameter of 220 nm was covered on the P-Si substrate (Fig. 1(a)). Second, a Pt film with 30 nm thickness was deposited on this sample by metal sputtering. After removing the PS spheres, the substrate was dry-etched in reactive ion etching (RIE) system by using 40 sccm CHF_3_ gas under RF power of 20 W(Fig. 1(b)). Then, the Pt NP-coated Si nanopillar array was obtained (Fig. 1(c)).

### (2) Fabrication process of embedded Si QDs/SiO_2_ multilayer

The amorphous Si (a-Si)/SiO_2_ multilayer film, composed of 8 pairs of 4 nm a-Si and 4 nm SiO_2_ layers, was prepared in the plasma-enhanced chemical vapor deposition (PECVD) system. During the deposition, the rf power and deposition temperature were kept at 35 W and 220^°^C. The deposition rate is about 0.1 nm/s under the reaction pressure 40 Pa. Silane with a flow rate of 8 sccm was used to deposit amorphous silicon and O_2_ with a flow rate of 25 sccm was used to oxidize the amorphous silicon layers. Then, the a-Si/SiO_2_ multilayer samples were dehydrogenated at 500 °C for 1 hour. After that, they were thermally annealed at 1100 °C for 1 hour to obtain Si QDs/SiO_2_ multilayer. Here, an 8-period multilayer film was deposited on Si substrate without nanopillar for comparison. To make electrodes for electroluminescence measurements, Al electrodes were evaporated on the bottom side and the thin ITO electrodes with 1.5 mm in diameter were deposited on the top.

### (3) Electroluminescence (EL), extinction and reflection spectra measurement

The measurement of the EL was performed on Fluoromax-2 spectroscopy (Jobin-Yvon) system by applying DC voltage. The spectrum of Pt was performed by using a Shimadzu UV-3600 spectrophotometer in the wavelength range from 200 nm to 700 nm. The typical illuminated area was chosen to be a pinhole with 5 mm diameter. The reflection spectra of front surface were measured by using Shimadzu UV-3600 spectrophotometer for nearly normal light incidence (5% offset) in the wavelength range from 200 nm to 1000 nm.

## Result and discussion

Figure 1(a) shows the image of PS spheres monolayer on substrate. It is a classic honeycomb structure.And the area can be as large as 2 × 2 cm^2^.After the sputtering of Pt, we removed all of the PS spheres. Then a very well-ordered triangular array of hexagonal Pt nanoparticles was obtained. It was found in Fig. 1(b).These Pt nanoparticles will be used as a mask in the following etching process. Figure 1(c) is an oblique-view SEM image of the Pt NPs-coated Si pillars. It is shown that periodic Si nanopillars can be obtained by transferring the patterns into silicon substrate. Figure 1(d) is the schematic diagram of electroluminescent structures.

**Figure 2:**
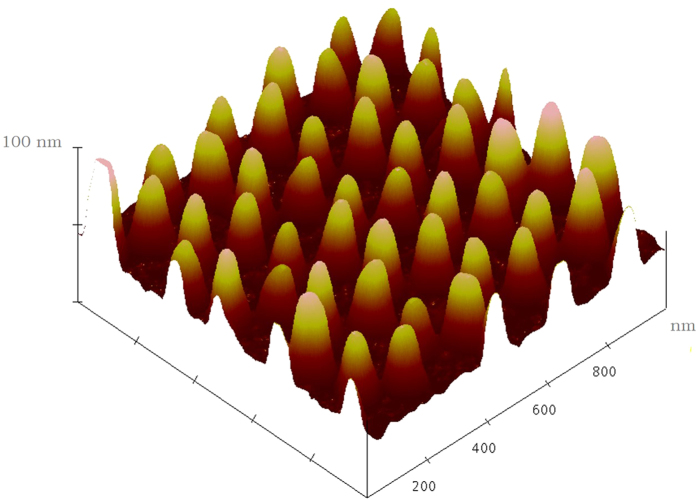
AFM of Pt nanoparticles coated Si nanopillar.

To get more information on the surface morphology, the samples were characterized by atomic force microscopy (AFM). Figure 2 shows the AFM image of embedded Si quantum dots /SiO_2_ film on Pt NP-coated Si nanopillar arrays substrate. As can be seen in this image, the pillars are quite uniform. The period constant is 220 nm which corresponds to the diameter of the PS spheres used here. The mean full width at half maximum (FWHM) of the nanopillar is estimated to be 50 nm, with 100 nm height.

**Figure 3:**
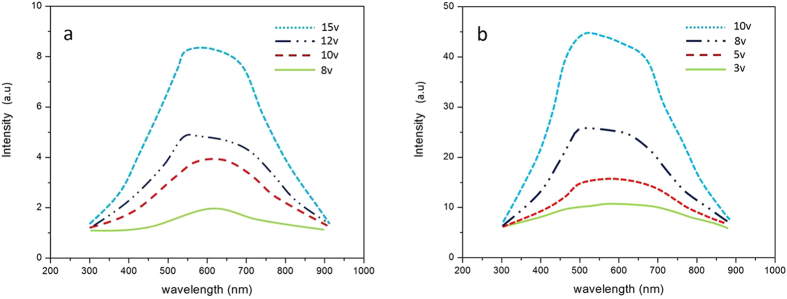
EL spectra of Si QDs/SiO_2_ (**a**) without Pt nanoparticles and Si nanopillar and (**b**) with Pt nanoparticles coated Si nanopillarsubstrate.

**Figure 4:**
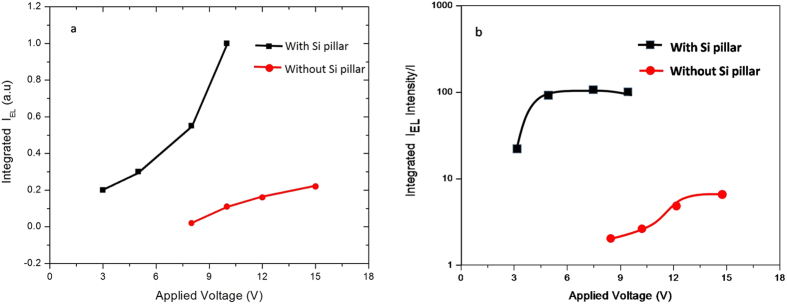
(**a**) Integrated EL intensity versus the applied DC voltage and (**b**) efficiency curves as a function of applied voltage .

**Figure 5:**
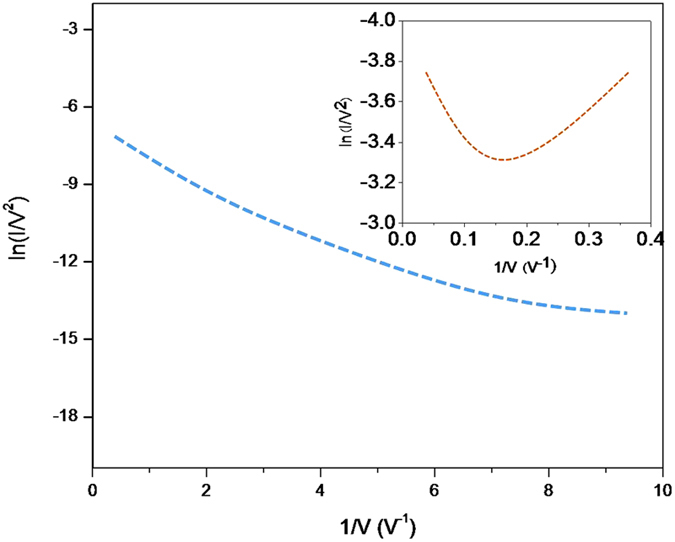
The curves of ln(I/V^2^) and 1/V for the samples with pillar. The inset is the curves for the sample without pillar.

**Figure 6:**
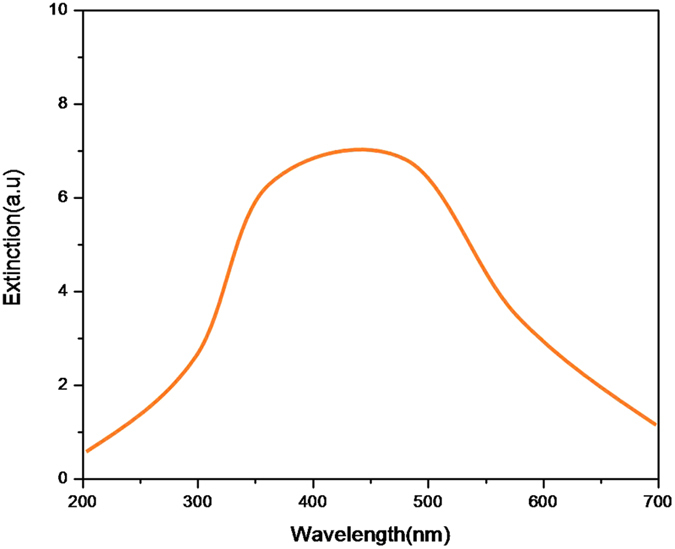
The extinction spectra of Pt nanoparticles.

Figure 3(a) and Fig. 3(b) show the EL spectra of a Si QDs/SiO_2_ multilayer film deposited on substrate without and with the Pt NP-coated nanopillar array. The EL signals were collected when applying DC voltage (from 3 V to15 V) and it shows that the EL band is quite broad. It looks like that the EL spectra of the sample without Si nanopillar contains at least two sub-bands located at 520 nm and 650 nm, respectively due to the different luminescence routes^22^,^23^,^24^,^25^.This spectra is from QCE in Si nanocrystals and defect-related EL bands in SiO_2_-based nanostructures. And these two bands also appear in the EL spectra of the sample with Si nanopillar. As observed from Fig. 3, the EL intensity increases with increased voltage. And the most important is that the EL intensity is obviously enhanced for the sample with Pt NPs-coated Si nanopillar compared to that without Pt NPs-coated Si nanopillar. Figure 4a depicts the normalized integrated EL intensities I_EL_ of the samples with and without Pt NPs-coated Si nanopillar as a function of applied voltage. The I_EL_ of both samples increases with increased voltage. When the applied voltage is 8 V or 10 V, the integrated EL intensities of the sample with Pt NPs-coated Si nanopillars are about 10 times stronger than that of without Pt NPs-coated Si nanopillars. Figure 4b shows the ratio of integrated EL intensity to the injection current as a function of applied voltage for both samples. The ratio of integrated EL intensity to the injection current directly reflects the external quantum efficiency. It is clearly shown that the external quantum efficiency is obviously enhanced for samples with Si pillar. The EL efficiency can be improved by almost two orders of magnitude as shown in Fig. 4(b). It was reported that the EL intensity can be improved due to the enhanced Fowler-Nordheim (F-N) tunneling process by introducing Si interfacial nano-pyramids^26^. The plots of ln (I/V^2^) vs 1/V for both samples are shown in Fig. 5. A nearly linear behavior for the samples with Si nanopillar indicates that the EL follows F-N tunneling mechanism in our case. Meanwile, it is found that the threshold voltage to initiate F-N tunneling is reduced obviously.The enhancement of the light emission is due to the resonance coupling between the LSP of Pt NPs. When the light emission wavelength of Si QDs/SiO_2_ multilayer is close to the extinction peak of Pt nanoparticles, this coupling process occurs. It is reported that the LSP energy of Pt NPs can be tuned in a wide range from the deep-UV to visible region by size control^27^,^28^. Figure 6 shows the extinction spectra of Pt nanoparticles. As expected, there is a wide extinction peak in this spectra. And, the FWHM of the peak is around 200 nm. In our researches, the Pt nanoparticle size is in the range of about 40 nm to 80 nm, shown in Fig. 1(b). This is the reason of the wide FWHM. Langhammer has reported that Pt nanoparticles have broader resonance peak and larger nonradiative damping than Au^29^,^30^. In our case, the peak of EL spectra from Si QDs is also wide (Fig. 3a). So, the LSP energy of Pt nanoparticle is close to the band-gap emission of the sample. Meanwhile, the larger nanoradiative damping make the more energy transfer from Pt nanoparticles to Si QDs. In addition, the better contract between Pt nanoparticles and Si QDs/SiO_2_ multilayer is formed after annealing. It is helpful for the LSP coupling.

**Figure 7:**
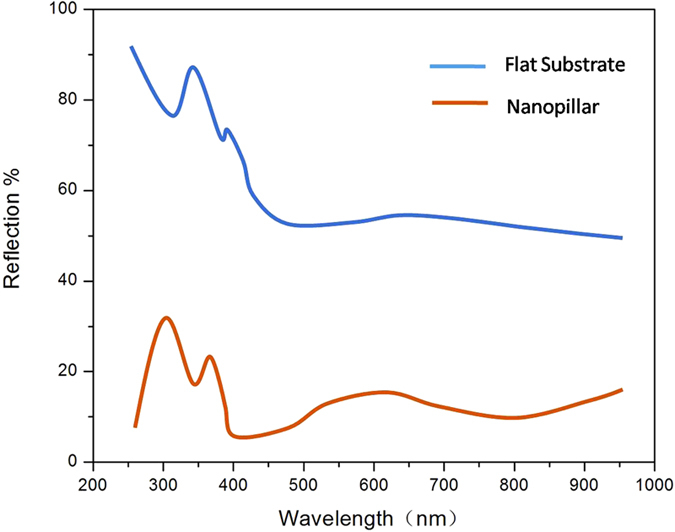
The reflection spectra for flat and nano-patterned Si substrates.

Another possible explanation for the EL enhancement factor would be the improved surface reflection by Si nanopillars as surface roughening structures. From Erchak’s paper, only 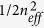
(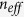 is the effective refraction index) of light can be extracted from the top^31^. Considering the index of Si and SiO_2_, which is about 3.4 and 1.5, it can be estimated that the light escaping from the flat sample without Pt NPs-coated Si nanopillar is 8.3%. As can be seen in Fig. 2, the Si nanopillars have a cone shape, which causes a gradually changing reflective index from top to bottom. And then, the 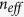 is reduced in the samples with Pt NPs-coated Si nanopillar, which leads to much more light emission. Figure 7 shows the reflection spectra for flat and nano-patterned Si substrates. It is shown that the reflectivity from the front surface of flat Si substrate is above 50% in the whole measurement range. And, it is interesting to find that the light reflection from the front surface is obviously suppressed for the nano-patterned samples, indicating the anti-reflection characteristics for nano-patterned Si substrates. It has been reported enhanced optical absorption was achieved from the Si-based nano-cone or nano-pillar structures and the antireflection^32^. So the light extraction efficiency can be significantly improved by surface roughing.

Moreover, the turn on voltage was different. Here, the turn on voltage is defined as the voltage at which the EL signal starts to be detected. The turn on voltage is about 8 V for the sample without Pt NPs-coated Si nanopillar. And it is reduced to 3 V for the sample with Pt NPs-coated Si nanopillar. It is well known that the sharp top of the pillar can lower the surface barrier. And the Pt NPs can supply more electrons. Both of them make the carrier injection into the Si QDs/SiO_2_ system easier, thus the EL intensity can be increased.

## Conclusion

In conclusion, we prepared an embedded Si quantum dot/SiO_2_ multilayer film on a Pt NPs-coated Si nanopillar array. It is found that the light extraction efficiency may be improved due to the LSP of Pt NPs and the surface roughing of Si nanopillars. As a consequence, the EL intensity is increased by 10 times compared with the flat substrate devices and the turn-on voltage is reduced to 3 V. Therefore, it is expected to have important applications in many areas of nanoscience and nanotechnology.

## Additional Information

**How to cite this article**: Li, W. *et al*. Enhancement of electroluminescence from embedded Si quantum dots/SiO_2_multilayers film by localized-surface-plasmon and surface roughening. *Sci. Rep*. **5**, 11881; doi: 10.1038/srep11881 (2015).
